# Leptospiral Hemolysins Induce Proinflammatory Cytokines through Toll-Like Receptor 2-and 4-Mediated JNK and NF-κB Signaling Pathways

**DOI:** 10.1371/journal.pone.0042266

**Published:** 2012-08-01

**Authors:** Huan Wang, Yifei Wu, David M. Ojcius, X. Frank Yang, Chenglin Zhang, Shibiao Ding, Xu’ai Lin, Jie Yan

**Affiliations:** 1 Department of Medical Microbiology and Parasitology, Zhejiang University School of Medicine, Hangzhou, Zhejiang, China; 2 Division of Basic Medical Microbiology, State Key Laboratory for Diagnosis and Treatment of Infectious Diseases, the First Affiliated Hospital of Zhejiang University School of Medicine, Hangzhou, Zhejiang, China; 3 Health Sciences Research Institute and Molecular Cell Biology, University of California, Merced, California, United States of America; 4 Department of Microbiology and Immunology, Indiana University School of Medicine, Indianapolis, Indiana, United States of America; National Council of Sciences (CONICET), Argentina

## Abstract

**Background:**

Infection with pathogenic *Leptospira* species causes serious systemic inflammation in patients. Although a few leptospiral proinflammatory molecules have been identified, *Leptospira* likely encodes other unidentified strong inflammation stimulators. The pathogenic *L. interrogans* genome encodes numerous putative hemolysin genes. Since hemolysins from other bacteria can cause inflammatory reactions, we hypothesized that leptospiral hemolysins may function as proinflammatory stimulators that contribute to the strong inflammation associated with *Leptospira* infection.

**Methodology/Principal Findings:**

We first used cytokine protein microarrays for systematic analysis of serum cytokine profiles in leptospirosis patients and leptospire-infected mice. We found that IL-1β, IL-6 and TNF-α were the main proinflammatory cytokines in the sera of both the patients and the mice. We then analyzed eight putative hemolysins in *L. interrogans* strain Lai. The results showed that five of them, Sph1, Sph2, Sph3, HlpA and TlyA were secreted and had hemolytic activity. More importantly, these five hemolysins induced the strong production of IL-1β, IL-6 and TNF-α in human and mouse macrophages (although a bit lower in the latter). Furthermore, blockade of TLR2 or TLR4 with either antibodies or inhibitors of the NF-κB or JNK signaling pathways significantly reduced the production of hemolysin-induced IL-1β, IL-6 and TNF-α. Macrophages isolated from TLR2-, TLR4-or double TLR2-and 4-deficient mice also confirmed that the leptospiral hemolysins that induce proinflammatory cytokines are both TLR2-and TLR4-dependent.

**Conclusions/Significance:**

Our findings demonstrate that *L. interrogans* secretes many hemolysins that function as powerful inducers of proinflammatory cytokines through both TLR2-and TLR4-dependent JNK and NF-κB pathways.

## Introduction

Leptospirosis is a worldwide zoonotic disease caused by pathogenic leptospires of the genus *Leptospira*. It has been identified as a neglected emerging infectious disease [1], [2]. Pathogenic *Leptospira* species can infect many animal species to cause a nearly asymptomatic infection [3]. After being shed in the urine of animals, the leptospires are able to survive for long periods in moist soil and natural bodies of water [4]. Transmission of the leptospires from animals to humans occurs through indirect contact with soil or water contaminated with animal urine [2]. The course of leptospirosis in humans varies from mild to rapidly fatal forms, including “flu-like” clinical manifestations such as high fever and myalgia, and severe cases with serious systemic inflammation, septic shock, jaundice, and multiple organ hemorrhage and failure, known as Weil’s syndrome [2]–[4].

Inflammation is an immunoprotective reaction during the early stages of infection mediated by proinflammatory factors from the host. The function of host inflammation is to eliminate the microbial pathogens. However, excessive production of proinflammatory agents also causes pathological inflammatory disorders and tissue injury [5]. In this regard, severe inflammatory symptoms occur in all leptospirosis patients [1], [3]. Earlier reports showed that TNF-α, an important proinflammatory cytokine produced mainly by mononuclear macrophages, is elevated in sera from leptospirosis patients [6], [7]. More recently, other cytokines, including IL-1, IL-6, IL-8, IL-10 and IL-12, were shown to be involved in inflammatory responses during *Leptospira* infection [8]–[11]. Two of the cytokines, TNF-α and IL-6, are strongly associated with the severity of disease and the mortality of leptospirosis [7], [11]. However, a complete profile of proinflammatory cytokines produced by hosts with leptospirosis as well as the leptospiral components that stimulate inflammation are poorly understood.

Several *Leptospira* components have been shown to trigger inflammatory reactions during infection. *Leptospira* possesses lipopolysaccharide (LPS), but its endotoxic potency is much lower than that of many other Gram-negative bacteria [12]. It has been shown that LPS from *Leptospira* is recognized by Toll-like receptor 2 (TLR2) alone in human THP-1 monocytes, but by both TLR2 and TLR4 in mouse RAW264.7 macrophages. This distinguishes it from most other bacterial LPSs that are recognized by TLR4 in both human and murine mononuclear macrophages [13]. Werts *et al.* reported that *Leptospira* LPS stimulates human THP-1 monocytes to produce IL-8 and TNF-α through a TLR2-dependent mechanism [14]. In addition to LPS, leptospiral peptidoglycan (L-PG) and glycolipoprotein (GLP) can also induce the release of TNF-α from human monocytes [15], [16]. Furthermore, a major leptospiral outer membrane lipoprotein, LipL32, was recently shown to induce early inflammation in human proximal tubule cells and animals [17]. Given the strong inflammatory response and tissue damage associated with the severe form of leptospirosis, however, it is likely that *Leptospira* contains additional unrecognized inflammation inducers.

Although pathogenic *Leptospira* species are highly pathogenic in humans, no exotoxin produced by the spirochete has been identified except for hemolysins [3], [18]. The genomes of *L. interrogans* serogroup Icterohaemorrhagiae serovar Lai strain Lai and serovar Copenhageni strain Fiocruz L1–130 (GenBank accession No.: NC_004342 and NC_005823) contain at least nine hemolysin-encoding genes [18], [19]. Among them, *sph1, sph2, sph3, sph4* and *sphH* were predicted to encode sphingomyelinase-type hemolysins, while *hlpA*, *hlyC*, *hlyX* and *tlyA* were postulated to encode non-sphingomyelinase hemolysins [18], [19]. However, relatively few of these putative hemolysin genes have been characterized. The *sphH* gene was shown to encode a pore-forming toxin against mammalian cells [20]. The *hlyC* (or *tlyC*) gene was reported to encode an extracellular matrix-binding protein without hemolytic activity [21]. The recombinant Sph2 protein was demonstrated to lyse sheep erythrocytes and to induce apoptosis in mouse lymphocytes and macrophages, as well as promote an increase in the levels of IL-1β and IL-6 *in vitro* [22]. However, the characteristics of the other putative leptospiral hemolysins have not been characterized. In addition to hemolytic activity, many hemolysins from other bacterial pathogens have been shown to be powerful inflammation inducers during infection. Gleason *et al*. reported that the α-hemolysin of *Escherichia coli* induces the release of high levels of IL-1β and TNF-α from murine macrophages [23]. Braun *et al*. found that pneumolysin, an α-hemolysin produced by *S. pneumoniae*, stimulates mouse macrophages to release IL-1, TNF-α and IL-6 [24]. Doran *et al*. demonstrated that the β-hemolysin of group B streptococcus promotes the secretion of IL-8 from mouse macrophages and contributes to the bacterial invasion of human lung epithelial cells [25]. Based on these reports, we postulated that leptospiral hemolysins may function as proinflammatory stimulators that contribute to the strong inflammation associated with *Leptospira* infection.

In this study, we first determined the complete profiles of proinflammatory cytokines in sera from leptospirosis patients and leptospire-infected mice using cytokine protein microarrays. We then characterized eight putative hemolysin genes in *L. interrogans* strain Lai, their secretion status, hemolytic activity, and more importantly, their ability to induce the production of major pro-inflammatory cytokines found during *Leptospira* infection in humans and mice. In addition, we further identified the TLRs and intracellular signaling pathways that are essential for regulating the expression of proinflammatory cytokines upon stimulation by leptospiral hemolysins.

## Materials and Methods

### Ethics Statement

All subjects gave written informed consent, and the study was approved by the Human Ethics Committee of the Medical School of Zhejiang University, and complied with the Declaration of Helsinki. Animal experiments were performed in accordance with the National Regulations for the Administration of Experimental Animals of China (1988-002) and the National Guidelines for Experimental Animal Welfare of China (2006-398). All the animal experimental protocols were approved by the Ethics Committee for Animal Experiment of Zhejiang University (Certificate No.: SCXK[zhe]2007-0030).

### Leptospiral Strain and Culture

*L. interrogans* serogroup Icterohaemorrhagiae serovar Lai strain Lai was provided by the National Institute for the Control of Pharmaceutical and Biological Products, Beijing, China. The leptospiral strain was cultured in Ellinghausen-McCullough-Johnson-Harris (EMJH) liquid medium at 28°C [26].

### Cell Lines and Culture

A human monocytic cell line (THP-1) and a mouse mononuclear macrophage-like cell line (J774A.1) were provided by the Cell Bank in the Institute of Cytobiology, Chinese Academy of Science, Shanghai, China. The cells were maintained in RPMI-1640 medium (Gibco, USA) supplemented with 10% fetal calf serum (FCS, Gibco), 100 U/mL penicillin (Sigma, USA) and 100 µg/mL streptomycin (Sigma) in an atmosphere containing 5% CO_2_ at 37°C. In particular, THP-1 cells were pre-treated with 10 ng/mL PMA (Sigma) at 37°C for 48 h to differentiate them into macrophages before use [27].

### Animals

Female C3H/HeJ mice (15±1 g, three weeks old), female C57BL/6 mice (18±2 g, four weeks old) and New Zealand white rabbits (3.0 to 3.5 kg) were provided by the Laboratory Animal Center of Zhejiang University. TLR2-or TLR4-or TLR2,4-deficient (TLR2^−/−^, TLR4^−/−^, or TLR2,4^−/−^) female C57BL/6 mice were kindly provided by Dr. L.Y. Shi (Medical College of Hangzhou Normal University, China).

### Sera of Leptospirosis Patients and Leptospire-infected Mice

Sera from three leptospirosis patients (males, aged 26, 29 and 30 years) within three days of disease onset were provided by the Center for Disease Prevention and Control of Zhejiang Province, China. The patients had a history of encounter with leptospire-contaminated water and typical clinical manifestations of leptospirosis, confirmed by the presence of visible leptospires in peripheral blood specimens by dark-field microscopy and subsequent fractional cultivation [3], [4]. All the leptospiral isolates from the patients were identified as belonging to *L. interrogans* serovar Lai by the microscopic agglutination test [12], [28].

Mouse infection experiments were based on previous reports [29]–[31]. To calculate the 50% lethal dose (LD_50_), C3H/HeJ mice were injected intraperitoneally with 10^4^, 10^5^, 10^6^, 10^7^ or 10^8^ cells of *L. interrogans* strain Lai in 0.5 mL EMJH liquid medium. The animals were monitored daily and the surviving animals were recorded within 7 days after challenge for calculation of LD_50_ using the Probit analysis [32]. The LD_50_ was ∼1×10^6^ leptospires similar to that determined in our previous study [33]. For serum collection, mice were intraperitoneally infected with 1×10^6^ *L. interrogans* Lai strain and peripheral blood samples from surviving mice were collected at 48 h after infection.

### Detection of Serum Cytokines by Protein Microarray

Cytokines in sera from the leptospirosis patients and leptospire-infected mice were detected using the quantitative RayBio® Human Inflammation Antibody Array 3.1 (Norcross, USA) and Mouse Inflammation Antibody Array 1.1 (Norcross). The two inflammation microarrays can detect 40 human and mouse cytokines including all the known proinflammatory cytokines. The controls for these assays were the pooled sera of three healthy volunteers (males, aged 26–30) and the pooled sera of three healthy C3H/HeJ mice after intraperitoneal injection with 0.5 mL EMJH liquid medium per animal.

### Expression and Purification of Recombinant Leptospiral Hemolysins

Genomic DNA of *L. interrogans* strain Lai was extracted using a Bacterial Genomic DNA Extraction Kit (BioColor, China). Several PCRs were performed to amplify each of the leptospiral *sph1*, *sph2*, *sph3*, *sph4*, *hlpA, hlyC, hlyX* and *tlyA* genes. The primer sequences are shown in **Table 1**. The products were detected on 1.5% ethidium bromide pre-stained agarose gels after electrophoresis, and then cloned into pMD18-T vector (TaKaRa, China) for sequencing by Invitrogen Co., Shanghai, China. The eight cloned genes with the expected sequences and pET42a plasmid (Novagen, USA) were digested with both *Nde* I and *Xho* I endonucleases (TaKaRa). Each of the recovered DNA segments of the target genes was linked with linearized pET42a using T4 DNA ligase (TaKaRa), and then transformed into *E. coli* BL21DE3 (Novagen). The engineered bacteria were cultured in kanamycin-containing Luria-Bertani liquid medium (pH_8.5; Oxoid, UK) to express soluble recombinant leptospiral hemolysins (rL-hemolysins) under induction by 0.5 mM isopropyl-β-D-thiogalactoside (IPTG, Sigma) at 25°C in a shaker with 150 rpm rotation for 10–12 h. Furthermore, the Protein Refolding kit (Novagen) was used to obtain more soluble rL-hemolysins according to the manufacturer’s protocol. The expressed rL-hemolysins were extracted using an Ni-NTA affinity chromatographic column (BioColor). The expression and purity of rL-hemolysins were determined by sodium dodecyl sulfate polyacrylamide gel electrophoresis (SDS-PAGE) plus Agarose Image Analyzer (Bio-Rad, USA).

**Table 1:** Sequences of the primers used in this study.

Primer	Sequence (5′ to 3′)	Target	Size (bp)
S1A	F: CGCCATATG(*Nde* I)CCTGGCAAAAAAAATTCCATA	*sph1* gene expression	1674
	R: CCGCTCGAG(*Xho* I)ATGATGATAGATTAAATC		
S1B	F: TTCCGACTCTTCAAATCCACG	*sph1*-mRNA detection	175
	R: TGCAATTCTTTGTGCCCTTTC		
S2A	F: CGCCATATG(*Nde* I)ATAAACAAAATAACA	*sph2* gene expression	1869
	R: CCGCTCGAG(*Xho* I)GCGATAAATAAGATCCGC		
S2B	F: GGTTGTGGAGCGGATTGGT	*sph2-*mRNA detection	104
	R: TCCTGAGACTGAGCGTGGG		
S3A	F: CGCCATATG(*Nde* I)GATTCATTAGTATACAATAAA	*sph3* gene expression	1557
	R: CCGCTCGAG(*Xho* I)ACGATAAATTAGATCCTTGC		
S3B	F: ACGACCCTGATTCAGACACCTT	*sph3-*mRNA detection	100
	R: CCTCCTCTAAGCCACCAAGTCC		
S4A	F: CGCCATATG(*Nde* I)AAAATGATACAAAATA	*sph4* gene expression	717
	R: CCGCTCGAG(*Xho* I)TTCCTCAGGGCCTTCATTCAA		
S4B	F: GCAGATCCTGAACGACCTGAC	*sph4-*mRNA detection	171
	R: TCCAAGCCACCAATTCCAATA		
HA1	F: CGCCATATG(*Nde* I)TCATTAGCTCCGATTGCA	*hlpA* gene expression	939
	R: CGCCTCGAG(*Xho* I)GAGCAAATTAGATTTGTC		
HA2	F: ACGAATACGATGTGGATTTGAGTT	*hlpA-*mRNA detection	185
	R: CTGGCCAAATTCTCGTTTTTATAC		
HC1	F: CGCCATATG(*Nde* I)GAATTAATCGGCTTTTTTATA	*hlyC* gene expression	1332
	R: CGCCTCGAG(*Xho* I)TTTTTTAGACTGAGCTCG		
HC2	F: GTTCACGGTTTGGCGATTACTAT	*hlyC-*mRNA detection	118
	R: ATAAAAAGTGCAATCGTTTCCGT		
HX1	F: CGCCATATG(*Nde* I)GTCGAAGCGCTGTCTGTC	*hlyX* gene expression	1176
	R: CGCCTCGAG(*Xho* I)ATCCAATTTTTCGGTTTC		
HX2	F: ATGGGAAGAGCACTTGAAAGAAAC	*hlyX-*mRNA detection	169
	R: TCTAAATGACCTTTTCCTTCTTCG		
TA1	F: CGCCATATG(*Nde* I)TTTCCACAGAAGAAGAAC	*tlyA* gene expression	828
	R: CGCCTCGAG(*Xho* I)TAAGTTCCAAAACAAAAG		
TA2	F: CTTCTACGGGAGGATTTACGCA	*tlyA-*mRNA detection	177
	R: ATAAAAAGTGCAATCGTTTCCGT		
16S	F: CTTTCGTCGCCTCAGCGTCAGT	I6 S rRNA as the inner	145
	R: CGCAGCCTGCACTTGAAACTA	reference in RT-qPCR	

F: forward primer. R: reverse primer.

### Removal of LPS from rL-hemolysin Proteins

Possible LPS contamination from *E. coli* BL21DE3 in the extracts of rL-hemolysins was removed with a Detoxi-Gel Endotoxin Removing Gel column (Thermo Scientific, USA) twice using pyrogen-free water for elution [34], [35]. The limulus test with a minimal detection sensitivity of 10 pg LPS was applied to detect *E. coli* LPS in the purified rL-hemolysins based on the manufacturer’s protocol (ACC, USA). Using sheep anti-*E. coli* LPS-IgG as the coated antibody, mouse anti-*E. coli* LPS-IgG as the primary antibody (Abcam, UK) and HRP-conjugated rabbit anti-mouse-IgG (Jackson ImmunoResearch, USA) as the secondary antibody, a sandwich ELISA was established for detection of LPS in the purified rL-hemolysins as previously described [36]. In the limulus test and ELISA, LPS of *E. coli* serotype O111:B4 (Sigma) was used as the control.

### Preparation of Antisera and IgGs

Rabbits were immunized intradermally on days 1, 7, 14 and 21 with each of the purified rL-hemolysins pre-mixed with Freund’s adjuvant. Fifteen days after the last immunization, the rabbit sera were collected to separate IgGs by ammonium sulfate precipitation plus a DEAE-52 column (Sigma) using 10 mM phosphate buffer (pH_7.4) for elution. The immunodiffusion test was applied to assess the titer of each of the IgGs binding the corresponding rL-hemolysin protein.

### Hemolytic Assays

The hemolytic activity of each of the rL-hemolysins was determined using a hemolytic assay based on spectrophotometric measurement as previously described [20], [21]. Briefly, a reaction mix (1 mL) containing 5% (vol/vol) sheep erythrocyte suspension and 20 mM MgCl_2_ in PBS was incubated with 1 or 10 µg of each of the purified rL-hemolysins for 90 min at 37°C. The mixtures were centrifuged at 12,000×g for 2 min. The hemoglobin in the supernatants was measured by spectrophotometry at 420 nm (A420). The A420 values in the supernatants from the same number of erythrocytes which were either lysed with 1 mL distilled water (total hemolysis) or treated with the same concentrations of a non-hemolytic recombinant porin (background hemolysis) from *L. interrogans* strain Lai (rOmpL1) [26] were also detected. The relative hemolytic activity of each of the rL-hemolysins was expressed as percent lysis (A420 of each sample – A420 of background/A420 of total – A420 of background) ×100 [37]. In the spectrophotometric hemolytic assay, 1 or 10 µg of each of the purified rL-hemolysins pretreated with 10 or 100 µg proteinase K (TaKaRa) at 55°C for 2 h followed by heating at 95°C for 10 min (PK-H/hemolysins) was used as the control [38]. The hemolytic assay on 2.5% sheep blood agar plates was performed using 10 µg of each of the rL-hemolysin proteins as previously described [21].

### Measurement of Leptospiral Hemolysin mRNA During Infection

Freshly cultured *L. interrogans* strain Lai was collected by 17,200×g centrifugation at 15°C for 15 min. The harvested leptospires were counted under a dark-field microscope with a Petroff-Hausser counting chamber (Fisher Scientific, USA) [39]. THP-1 or J774A.1 cells (1×10^6^ per well) were seeded in 6-well culture plates (Corning, USA) for incubation in an atmosphere of 5% CO_2_ at 37°C for 24 h. The cell monolayers were washed with PBS and then infected with the harvested leptospires (1×10^8^) at a multiplicity of infection (MOI) of 100 (100 leptospires per host cell) for 0.5, 1, 2, 4, 8, 12, 24 or 48 h [40]. The cultures were treated with 0.05% sodium deoxycholate to lyse the cells [33], and then centrifuged at 17,200×g for 15 min at 4°C to precipitate the leptospires. Total leptospiral RNA was extracted using TRIzol reagent (Invitrogen) and then treated with RNase-free DNase using a DNA Eraser Kit (TaKaRa) to remove residual DNA according to the manufacturers’ protocols. cDNAs from the RNAs were synthesized by reverse transcription (RT) using an M-MLV RTase cDNA Synthesis Kit (TaKaRa). Using the cDNAs as the templates, mRNAs of the eight leptospiral hemolysin-encoding genes were measured by real-time fluorescence quantitative PCR (qPCR) using a SYBR® Premix Ex-Taq™ II Kit (TaKaRa) in an ABI 7500 Real-Time PCR System (ABI, USA). The primers used in the qPCR are shown in **Table 1**. For the qPCR, the 16 S rDNA gene of *L. interrogans* strain Lai was used as the internal reference [41]. The qPCR data were analyzed using the ΔΔCt model and randomization test in REST 2005 software [42].

### Determination of Secretion of Leptospiral Hemolysins

THP-1 or J774A.1 cells (1×10^7^) were infected with *L. interrogans* strain Lai (1×10^9^) at an MOI of 100 for 1, 2, 4 or 8 h at 37°C. Supernatants of the co-incubation cultures and *L. interrogans* strain Lai culture in EMJH liquid medium were collected by 17,200×g centrifugation at 4°C for 15 min, and then trichloroacetic acid (TCA) was added to a final concentration of 10% (vol/vol) for 60 min incubation on ice. After 17,200×g centrifugation at 4°C for 15 min, the leptospiral precipitates were washed twice with ice-cold acetone to remove TCA. The precipitates were dissolved in 1 ml distilled water for SDS-PAGE and then electrotransferred onto PVDF membranes. Using each of the 1∶100 diluted rabbit anti-hemolysin-IgGs as the primary antibody and 1∶3,000 diluted HRP-conjugated goat anti-rabbit-IgG (Jackson ImmunoResearch) as the secondary antibody, several Western blot assays were performed to detect the leptospiral hemolysins in the protein extracts from the supernatants [43]. To obtain reliable results, each experimental step was strictly quantitative. In the assays, the normal rabbit IgG (Sigma) as the primary antibody was used as the control. Furthermore, FliY, a protein component of leptospiral flagellar basal body with intracellular location, was selected as another control for rL-hemolysin secretion detection, and the rabbit anti-rFliY-IgG was provided by our laboratory [44].

**Figure 1:**
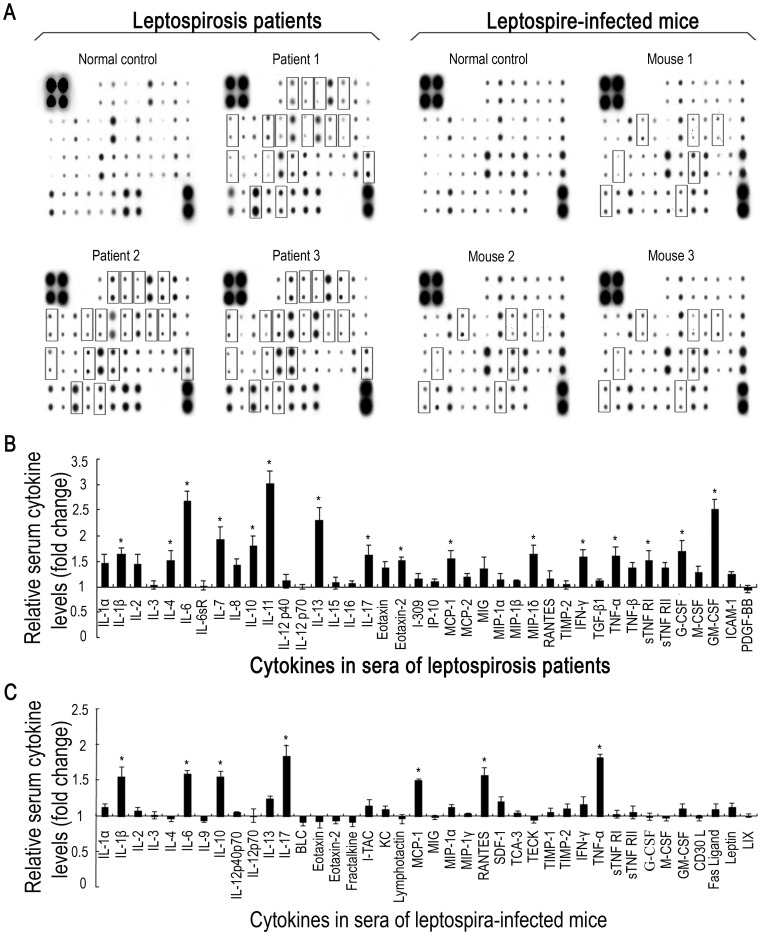
Cytokine profiles in sera from leptospirosis patients and leptospire-infected mice. (A). Protein array detection of cytokines in sera from leptospirosis patients within three days after disease onset and C3H/HeJ mice infected with *L. interrogans* Lai strain for 48 h. The hybridization blots within squares indicate the cytokines with significant elevation (≥1.5 fold) in all the serum specimens from leptospirosis patients compared to that in the pooled sera from three healthy individuals, or from leptospire-infected mice compared to that in pooled sera from three uninfected mice. (B). Statistical summary of the cytokine profile of the sera from leptospirosis patients. Statistical data from the cytokine array detection such as shown in A (leptospirosis patients). Bars show the levels of different cytokines with mean ± SD in the sera of three leptospirosis patients. The levels of cytokines in pooled serum from three healthy individuals were set as 1.0. *: cytokine level significantly elevated (≥1.5 fold) compared to that in the pooled serum from three healthy individuals. (C). Statistical summary of the cytokine profile of the sera from leptospire-infected mice. Statistical data from the cytokine array such as shown in A (leptospire-infected mice). Bars show the levels of different cytokines with mean ± SD in the sera of three leptospire-infected mice. The levels of cytokines in pooled serum from three uninfected mice were set as 1.0. *: the cytokine level significantly elevated (≥1.5 fold) compared to that in the pooled serum from three uninfected mice.

### Detection of Leptospiral Secretory Hemolysin-induced Proinflammatory Cytokines

THP-1 or J774A.1 cells (1×10^5^ per well) were seeded on 24-well culture plates (Corning), and then incubated in an atmosphere of 5% CO_2_ at 37°C for 24 h. The cell monolayers were treated at 37°C for 24 h with 0.1, 1 or 10 µg of each the secretory rL-hemolysin proteins (rSph1, rSph2, rSph3, rHlpA and rTlyA) or the same concentrations of each the five rL-hemolysins pretreated with proteinase K plus heating as described above (PK-H/hemolysins) or with 2 µg polymyxin B sulfate (Invitrogen) at 37°C for 30 min (PMB/hemolysins) [45]. After centrifugation at 500×g for 10 min (4°C), the supernatants from the rL-hemolysin-treated cultures were collected to detect the IL-1β, IL-6 and TNF-α levels using quantitative human or mouse cytokine ELISA kits (eBioscience, USA) according to the manufacturer’s protocol. On the other hand, the two cell types were treated with 1 µg of each of the five rL-hemolysin proteins at 37°C for 1, 2, 6, 12, 24, 48 or 72 h, followed by the detection of IL-1β, IL-6 and TNF-α in the supernatants as described above. In the ELISAs for cytokine detection, LPS of *E. coli* serotype O111:B4 (E-LPS) (Sigma), and E-LPS pretreated with proteinase K plus heating (PK-H/E-LPS) or PMB (PMB/E-LPS) as above were used as the controls. Viability of the rL-hemolysin-treated cells was monitored using a CCK-8 agent (Kumamoto, Japan), and the experimental steps and the calculation of percentage cell viability were performed following the manufacturer’s instructions.

### Determination of Leptospiral Hemolysin-recognizing TLRs

THP-1 or J774A.1 cells (1×10^5^ per well) were seeded in 24-well culture plates (Corning), and then incubated in an atmosphere of 5% CO_2_ at 37°C for 24 h. The cell monolayers were pre-blocked with 1∶100 diluted rabbit anti-human or anti-mouse TLR1-, 2-, 4-, 5-or 6-IgG (Santa Cruz, USA) for 60 min at 37°C. After washing with PBS, the cells were treated with 1 µg of each of the rL-hemolysin proteins or the same concentrations of each the five rL-hemolysins (PK-H/hemolysins) pretreated with proteinase K plus heating or polymyxin B (PMB/hemolysins) as above for 24 h at 37°C. The IL-1β, IL-6 and TNF-α levels in the supernatants of cultures were determined as described above. TLR2-, TLR4-or TLR2,4-deficient monocytes were separated from peripheral blood of the TLR2^−/−^, TLR4^−/−^ or TLR2,4^−/−^ C57BL/6 mice using standard Ficoll-Hypaque gradient centrifugation plus CD11b-immunomagnetic beads (Miltenyi-Biotec, Germany) [46]. Treatment of the monocytes with each of the rL-hemolysins and detection of IL-1β, IL-6 and TNF-α levels in the supernatants of monocyte cultures were the same as described above. In this assay, the monocytes from peripheral blood of wild-type C57BL/6 mice, LPS of *E. coli* serotype O111:B4 (E-LPS) (Sigma) and E-LPS pretreated with proteinase K plus heating (PK-H/E-LPS) or PMB (PMB/E-LPS) as above were used as the controls.

### Blocking Test of Inflammation-associated Intracellular Signaling Pathways

THP-1 or J774A.1 cells (1×10^5^ per well) were seeded in 24-well culture plates (Corning), and then incubated in an atmosphere of 5% CO_2_ at 37°C for 24 h. The cell monolayers were pre-blocked with 20 µM mitogen-activated protein kinase 38 (p38MAPK) inhibitor SB203580 (Tocris Bioscience, USA), c-Jun N-terminal kinase (JNK) inhibitor SP600125 (Tocris Bioscience) or nuclear factor κ-B (NF-κB) inhibitor SN50 (Tocris Bioscience) for 60 min at 37°C [47], and then treated with 1 µg of each the rL-hemolysin proteins or the same concentrations of each the five rL-hemolysins pretreated with proteinase K plus heating (PK-H/hemolysins) or polymyxin B (PMB/hemolysins) as described above for 24 h at 37°C. The IL-1β, IL-6 and TNF-α levels in the supernatants of cultures were determined as above. In this assay, LPS of *E. coli* serotype O111:B4 (E-LPS) (Sigma), and the LPS pretreated with proteinase K plus heating (PK-H/E-LPS) or PMB (PMB/E-LPS) were used as the controls.

### Statistical Analysis

Data from at least three independent experiments were averaged to present as mean ± SD (standard deviation). One-way analysis of variance (ANOVA) followed by Dunnett’s multiple comparisons test were used to determine significant differences. Statistical significance was defined as *P*≤0.05.

**Figure 2:**
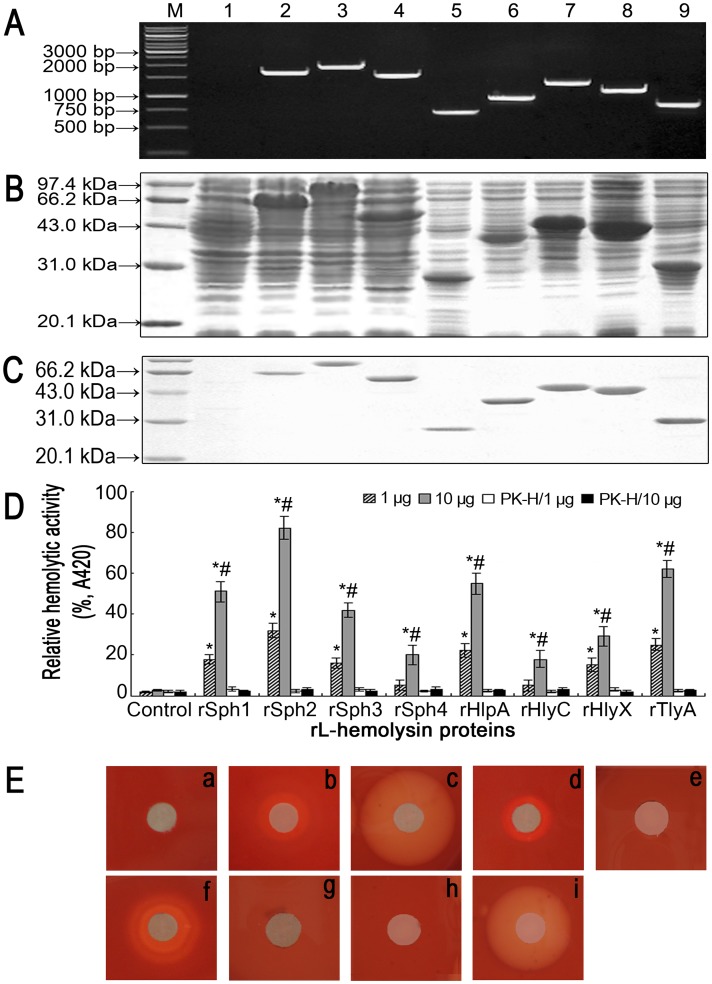
Expression, purification and hemolytic activity of rL-hemolysin proteins. (A). Hemolysin genes amplified from genomic DNA of *L. interrogans* strain Lai. Lane AM: DNA marker (Fermentas, Canada). Lane A1: blank control. Lanes A2 to A9: amplicons of the *sph1* (1674 bp), *sph2* (1869 bp), *sph3* (1557 bp), *sph4* (717 bp), *hlpA* (939 bp), *hlyC* (1332 bp), *hlyX* (1176 bp) and *tlyA* (828 bp) genes from *L. interrogans* strain Lai. (B). Expression of the rL-hemolysin proteins. Lane BM: protein marker (Sangon Biotech, China). Lane B1: wild-type pET42a. Lanes B2 to B9: expressed recombinant proteins of rSph1 (63.5 kDa), rSph2 (71.1 kDa), rSph3 (60.7 kDa), rSph4 (27.9 kDa), rHlpA (36.5 kDa), rHlyC (50.4 kDa), rHlyX (43.1 kDa) and rTlyA (31.5 kDa). (C). Purification of the rL-hemolysin proteins. Lane CM: protein marker (Sangon Biotech). Lane C1: blank control. Lanes C2 to C9: purified rSph1, rSph2, rSph3, rSph4, rHlpA, rHlyC, rHlyX and rTlyA proteins. (D). Hemolytic activity of the rL-hemolysin proteins measured by spectrophotometry. Bars show the mean ± SD of three independent experiments. PK-H indicates that the rL-hemolysins were pretreated with proteinase K (PK) digestion plus heat-inactivation. The A420 values from spectrophotometric measurement at 420 nm reflect the levels of hemoglobin released from sheep erythrocytes. The A420 value of the supernatant from the sheep erythrocytes in 1 mL 5% erythrocyte suspension lysed by distilled water was set at 100% (total hemolysis). rOmpL1, a non-hemolytic recombinant porin from *L. interrogans* strain Lai, was the negative control (background hemolysis). Relative hemolytic activity of each of the rL-hemolysin proteins was defined as the percentage (%, A420) compared to total hemolysis. *P<0.05 *vs* relative hemolytic activity of leptospiral rOmpL1 protein, the negative control, at the same protein concentrations. ^#^ *P*<0.05 *vs* the relative hemolytic activity of 1 µg of the corresponding rL-hemolysin protein.(E). Hemolytic rings on sheep blood agar plates caused by the rL-hemolysin proteins. a: negative control containing 10 µg rOmpL1, a non-hemolytic recombinant porin from *L. interrogans* strain Lai. b to i: hemolytic rings caused by the rL-hemolysin proteins rSph1, rSph2, rSph3, rSph4, rHlyC, rHlyX, rHlpA and rTlyA.

## Results

### Profiles of Cytokines in Sera of Leptospirosis Patients and Leptospire-infected Mice

Leptospirosis is characterized by the development of vasculitis, endothelial damage, and strong inflammatory infiltrates. However, a complete serum cytokine profile of human leptospirosis has not been previously reported. Thus, we used a cytokine protein array to determine the cytokine profiles in sera from leptospirosis patients during the acute phase of infection. The results revealed at least 16 highly expressed cytokines in the leptospirosis patients’sera (**Figure 1A and 1B**). Among the sixteen elevated cytokines, eight were proinflammatory factors (IL-1β, IL-6, IL-17 and TNF-α) and antiinflammatory factors (IL-4, IL-10, IL-13 and sTNF RI), while the others were immunoregulators (IL-7, IL-11 and IFN-γ), colony-stimulating factors (G-CSF and GM-CSF), or chemotactic factors (MCP-1, MIP-1δ and EOTAXIN-2). However, the mice infected with a sub-lethal dose of *L. interrogans* strain Lai displayed much less elevated serum cytokines during the acute phase of infection, including four proinflammatory factors (IL-1β, IL-6, IL-17 and TNF-α), an anti-inflammatory factor (IL-10) and two chemotactic factors (MCP-1 and RANTES) (**Figure 1A and 1C**). All these data suggest such serum cytokine elevation in both patients and mice during acute infection of *Leptospira*, and this should be confirmed in future with a larger analysis.

### Characterization of the rL-hemolysins

So far few bacterial factors that induce strong inflammatory responses have been identified in *Leptospira*. Because the pathogenic *Leptospira* genomes possess large numbers of putative hemolysin-encoding genes, and the hemolysins from other bacteria have been shown to induce the production of inflammatory cytokines [18], [19], [23]–[25], we therefore focused on the eight leptospiral hemolysin-encoding genes (*sph1*, *sph2*, *sph3*, *sph4*, *hlpA*, *hlyC*, *hlyX* and *tlyA*). The PCR results showed that all eight were present in the genomic DNA of *L. interrogans* strain Lai (**Figure 2A**). The recombinant leptospiral hemolysin proteins (rSph1, rSph2, rSph3, rSph4, rHlpA, rHlyC, rHlyX and rTlyA) were expressed in the pET42a-*E. coli* BL21DE3 system (**Figure 2B**), and each of the purified rL-hemolysins showed a single band in gels after SDS-PAGE (**Figure 2C**). The results from both the limulus test and LPS-ELISA confirmed that no *E. coli* LPS was detectable in any of the rL-hemolysin extracts with at least 100 µg protein/mL.

### Hemolytic Activity of the rL-hemolysins

The spectrophotometry-based hemolytic assays showed that 1 or 10 µg of rSph1, rSph2, rSph3, rHlpA, rHlyX and rTlyA, as well as 10 µg of rSph4 and rHlyC had different degrees of hemolytic activity (**Figure 2D**). However, rSph1, rSph2, rSph3, rHlpA and rTlyA, but not rSph4, rHlyC and rHlyX, displayed hemolytic rings on sheep blood agar plates (**Figure 2E**).

### Expression and Secretion Profiles of the Leptospiral Hemolysins

**Figure 3:**
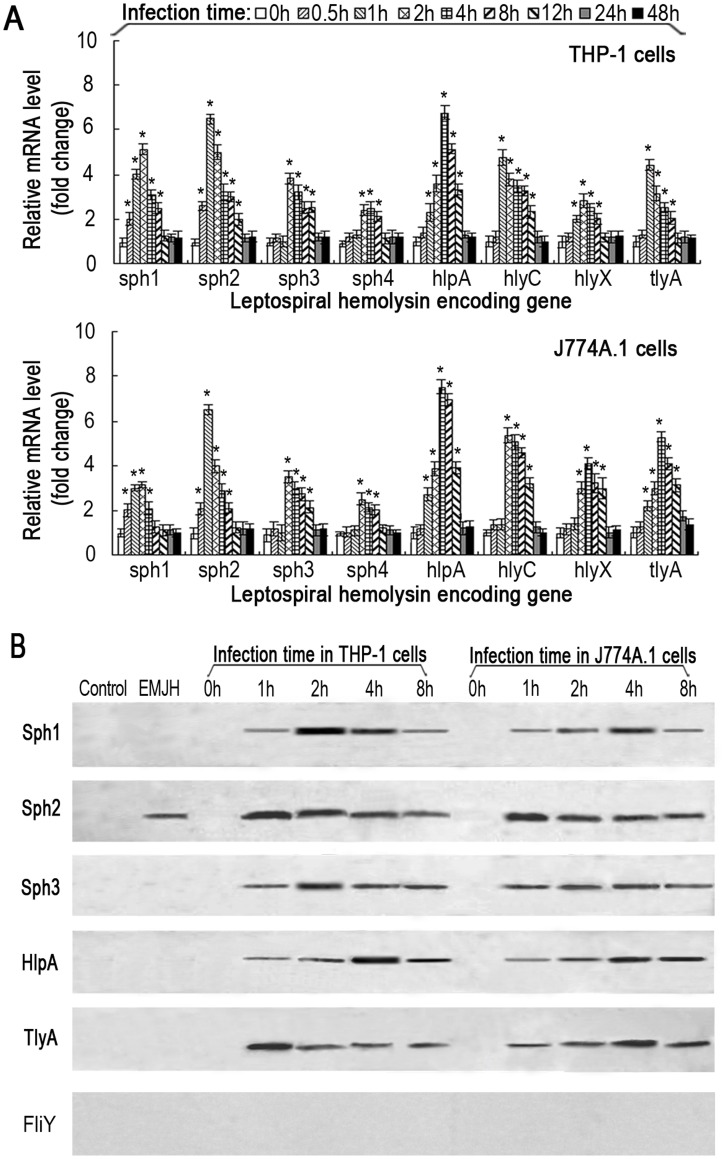
Up-regulation of mRNA levels and secretion of leptospiral hemolysins during infection of host cells. (A). Increase of mRNA levels of the leptospiral hemolysin-encoding genes in human THP-1 or mouse J774A.1 macrophages infected with *L. interrogans* strain Lai for the indicated times. Bars show the mean ± SD of three independent experiments. The values at 0 h indicate the mRNA levels of *sph1*, *sph2*, *sph3*, *sph4*, *hlpA, hlyC, hlyX* and *tlyA* genes before infection with the spirochete. *P<0.05 *vs* mRNA levels of the corresponding hemolysin-encoding genes of *Leptospira* strain Lai before infection of THP-1 or J774A.1 macrophages. (B). Secretion of the hemolysin proteins from *L. interrogans* strain Lai in medium or during infection of host cells. For control, normal rabbit IgG replaced the rabbit anti-rL-hemolysin-IgGs as the primary antibody. EMJH means that the TCA-precipitated protein specimens were extracted from the supernatants of leptospires cultured in EMJH medium. The 0 h lane indicate the immunoblot results of the TCA-precipitated protein specimens from the supernatants of human THP-1 or mouse J774A.1 macrophages before infection with the spirochete. The positive immunoblotting bands show the secreted leptospiral hemolysins (Sph1, Sph2, Sph3, HlpA and TlyA). FliY is a protein component of leptospiral flagellar basal body with intracellular location which used as the control for rL-hemolysin secretion detection.

Secretion is important for bacterial hemolysins to play roles in pathogenicity during infection. We therefore determined the expression and secretion profiles of *L. interrogans* strain Lai hemolysins during infection of host cells. The mRNA levels of *sph1*, *sph2*, *sph3*, *sph4*, *hlpA*, *hlyC*, *hlyX* and *tlyA* in the spirochete grown in EMJH medium were relatively low. Upon co-incubation of the spirochete with human THP-1 or mouse J774A.1 macrophages for 24 h, the mRNA levels for all the leptospiral hemolysin-encoding genes examined were significantly elevated (**Figure 3A**). These results suggest that the expression of leptospiral hemolysin genes are induced upon infection.

To determine whether these hemolysins are secreted by *L. interrogans* strain Lai, the spirochete was cultivated in EMJH medium or co-incubated with THP-1 or J774A.1 cells. The supernatants were collected for TCA precipitation, and then the protein extracts were subjected to immunoblot analysis. The results showed that only the Sph2 protein was detectable in the supernatant collected from the EMJH medium, while the Sph1, Sph2, Sph3, HlpA and TlyA proteins were readily detectable in the supernatant from co-incubation cultures of *L. interrogans* strain Lai with either THP-1 or J774A.1 cells (**Figure 3B**). The Sph4, HlyC and HlyX proteins were not detected in any of the supernatants, suggesting that these proteins are not secreted.

### Inflammatory-inducing Ability of the Leptospiral Hemolysins

**Figure 4:**
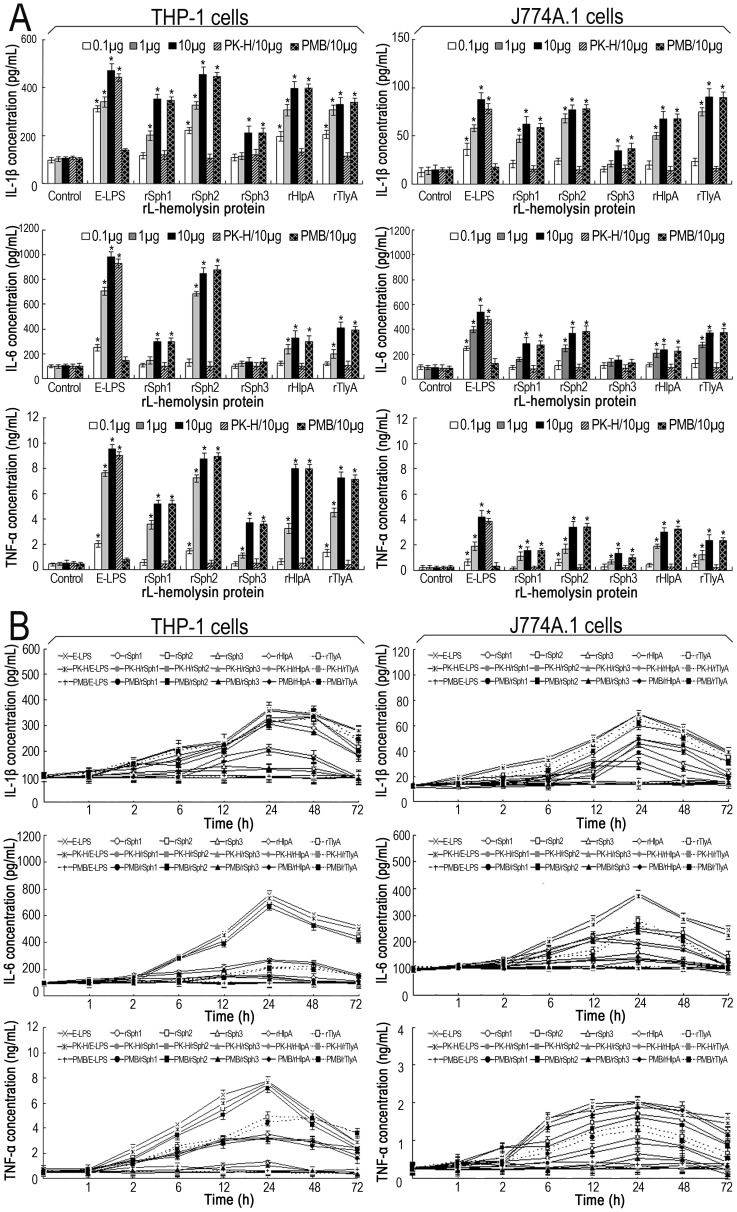
Ability of the rL-hemolysin proteins to induce IL-1β, IL-6 and TNF-α in human and mouse macrophages. (A). IL-1β, IL-6 and TNF-α levels in human THP-1 and mouse J774A.1 macrophages induced by each of the rL-hemolysin proteins for 24 h with the indicated protein concentrations. Bars show the mean ± SD of three independent experiments. E-LPS indicates the LPS of *E. coli* serotype O111:B4. PK-H indicates that the rL-hemolysins or E-LPS were pretreated with proteinase K digestion plus heat-inactivation while PMB indicates that the rL-hemolysins or E-LPS were pretreated with polymyxin B blockade, and used as controls to monitor possible contamination with *E. coli* LPS in the rL-hemolysin proteins. Controls indicate the IL-1β, IL-6 and TNF-α levels in the THP-1 or J774A.1 macrophages before treatment with any rL-hemolysins or E-LPS. E-LPS indicates the LPS of *E. coli* serotype O111:B4. *P<0.05 *vs* IL-1β, IL-6 and TNF-α levels in the THP-1 or J774A.1 macrophages before treatment with any rL-hemolysins or E-LPS (control). (B). Elevation patterns of IL-1β, IL-6 and TNF-α in rL-hemolysin-treated human THP-1 and mouse J774A.1 macrophages for the indicated co-incubation times. The data show the mean ± SD of three independent experiments. E-LPS indicates the LPS of *E. coli* serotype O111:B4. PK-H indicates that the rL-hemolysins or E-LPS were pretreated with proteinase K digestion plus heat-inactivation while PMB indicates that the rL-hemolysins or E-LPS were pretreated with polymyxin B blockade, and were used to monitor possible contamination with *E. coli* LPS in the rL-hemolysin proteins. The concentration of each of the rL-hemolysins and E-LPS tesed was 1 µg. The values at the 0 h shows the IL-1β, IL-6 and TNF-α levels in the THP-1 and J774A.1 macrophages before treatment with any rL-hemolysins or E-LPS.

To determine whether leptospiral hemolysins stimulate inflammatory responses, THP-1 or J774A.1 cells were treated with each of the five rL-hemolysin proteins (rSph1, rSph2, rSph3, rHlpA and rTlyA) that were confirmed to be secreted by *L. interrogans* strain Lai during infection of host cells. None of the rL-hemolysin proteins affected the viability of THP-1 or J774A.1 cells at the doses tested (0.1, 1 or 10 µg rL-hemolysin protein per 1×10^5^ cells; data not shown). All the rL-hemolysin-treated THP-1 or J774A.1 cells secreted high levels of IL-1β and TNF-α in a dose-dependent manner (**Figure 4A**). Except for the rSph3 protein, all the other rL-hemolysin proteins also induced high levels of IL-6 secretion from the two types of host cells (**Figure 4A**). In addition, the Sph2 protein stimulated the highest levels of cytokine production, and the rL-hemolysin-treated J774A.1 cells secreted significantly lower levels of the cytokines than the rL-hemolysin-treated THP-1 cells (**Figure 4A**). Furthermore, the highest levels of IL-1β, IL-6 and TNF-α secretion by both the THP-1 and J774A.1 cells occurred at 24 h of incubation with each of the rL-hemolysins (**Figure 4B**). More importantly, the levels of cytokine induction by the rSph2, rHlpA and rTlyA proteins were close to that induced by the LPS from *E. coli* (**Figure 4A and B**). In particular, polymyxin B treatment did not erase the cytokine-inducing ability of the rL-hemolysins whereas the proteinase K plus heating treatment made the cytokine-inducing ability of the rL-hemolysins disappear. These results suggest that the leptospiral hemolysins are potent inflammatory stimulants.

### Recognition of the Leptospiral Hemolysins Through TLR2 and TLR4

**Figure 5:**
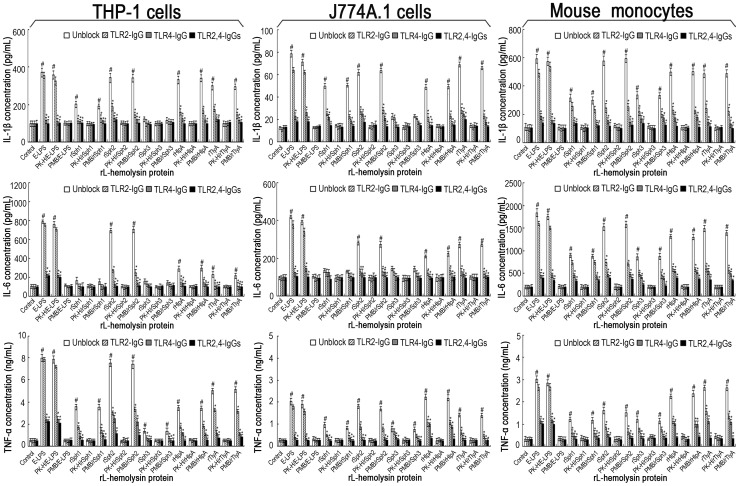
Blocking effects of TLR-IgGs and TLR2 or TLR4 deficieincy on the production of IL-1β, IL-6 and TNF-α under induction of the rL-hemolysins for 24 h. Bars show the mean± SD of three independent experiments. E-LPS indicates the LPS of *E. coli* serotype O111:B4. PK-H indicates that the rL-hemolysins or E-LPS were pretreated with proteinase K digestion plus heat–inactivation while PMB indicates that the rL-hemolysins or E-LPS were pretreated with polymyxin B blockade, and were used to monitor possible contamination with *E. coli* LPS in the rL-hemolysin proteins. The concentration of each of the rL-hemolysins or E-LPS tesed was 1 µg. The mouse monocytes were separated from the peripheral blood samples from TLR2^−/−^, TLR4^−/−^ or TLR2,4^−/−^ C57BL/6 mice. Control indicates the IL-1β, IL-6 and TNF-α levels in the human THP-1, mouse J774A.1 macrophages, and primary mouse monocytes from wild-type C57BL/6 without TLR2-, TLR4-or TLR2,4-deficience before treatement with any rL-hemolysins or E-LPS. ^#^ *P*<0.05 *vs* IL-1β, IL-6 and TNF-α levels in the THP-1 or J774A.1 macrophages or primary mouse monocytes before treatment with any rL-hemolysins or E-LPS (control). *P<0.05 *vs* IL-1β, IL-6 and TNF-α levels in the THP-1 or J774A.1 macrophages that had not been blocked with the TLR2,and/or TLR4 antibodies, or *vs* the primary monocytes from wild-type C57BL/6 without TLR2-, TLR4-or TLR2,4-deficiency.

We next investigated whether any TLRs play a role in signaling the leptospiral hemolysin-induced cytokine production. The results showed that TLR1-, TLR5-and TLR6-IgG failed to block the release of IL-1β, IL-6 and TNF-α in the THP-1 and J774A.1 cells treated with each of the five rL-hemolysins (rSph1, rSph2, rSph3, rHlpA or rTlyA) (data not shown), suggesting that TLR1, TLR5, and TLR6 are not required for the signaling. In contrast, TLR2-or TLR4-IgG significantly inhibited the cytokine production by THP-1 and J774A.1 cells upon rL-hemolysin stimulation, and combining both TLR2-IgG and TLR4-IgG provided stronger inhibition (**Figure 5**). Furthermore, TLR2-or TLR4-deficient monocytes isolated from TLR2^−/−^ or TLR4^−/−^ mice displayed significantly lower levels of IL-1β, IL-6 or TNF-α production upon rL-hemolysin stimulation, while TLR2,4 double-deficient monocytes isolated from TLR2,4^−/−^ mice failed to respond to any of the rL-hemolysins tested (**Figure 5**). Combined with the results of the rL-hemolysins pretreated with proteinase K digestion plus heating activation or polymyxin B blockage, all the data indicate that both TLR2 and TLR4 are the receptors for the leptospiral hemolysins.

### Leptospiral Hemolysin-induced Proinflammatory Cytokine Production Through NF-κB and JNK Pathways

**Figure 6:**
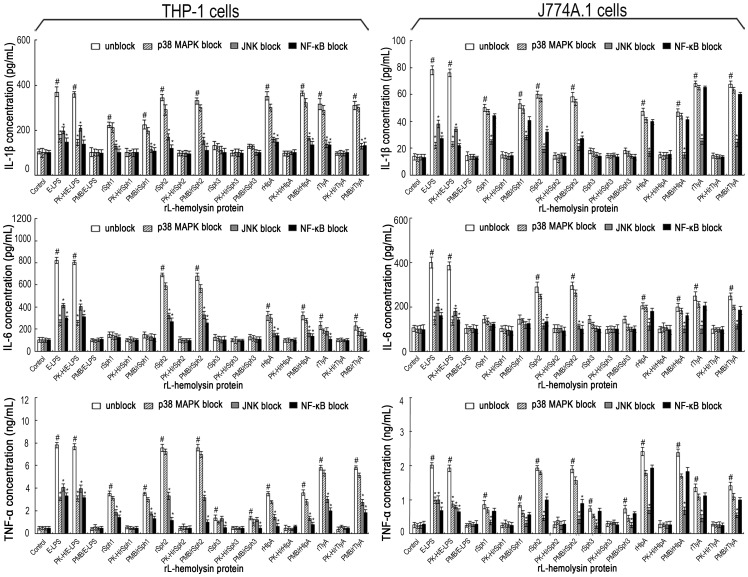
JNK and NF-κB pathways mediate the production of IL-1β, IL-6 and TNF-α under induction of the rL-hemolysins for 24 h. Bars show the mean ± SD of three independent experiments. E-LPS indicates the LPS of *E. coli* serotype O111:B4. PK-H indicates that the rL-hemolysins or E-LPS were pretreated with proteinase K digestion plus heat-inactivation while PMB indicates that the rL-hemolysins or E-LPS were pretreated with polymyxin B blockade, and were used to monitor possible contamination with *E. coli* LPS in the rL-hemolysin proteins. The concentration of each of the rL-hemolysins or E-LPS tesed was 1 µg. Control indicates the IL-1β, IL-6 and TNF-α levels in the human THP-1 and mouse J774A.1 macrophages before treatment with any rL-hemolysins or E-LPS. ^#^ *P*<0.05 *vs* IL-1β, IL-6 and TNF-α levels in the THP-1 or J774A.1 macrophages before treatment with any rL-hemolysins or E-LPS (control). *P<0.05 *vs* IL-1β, IL-6 and TNF-α levels in the THP-1 or J774A.1 macrophages that had not been treated with different signaling pathway inhibitors (SN50 for the NF-κB pathway, SP600125 for the JNK pathway, and SB203580 for the p38MAPK pathway).

It is known that interaction of a ligand with TLR2 or TLR4 activates cytokine encoding gene transcription through NF-κB, JNK or p38 signaling pathways [48]. To determine which pathway is required for the leptospiral hemolysin-induced cytokine production, different inhibitors were added to the reaction. The results showed that both an NF-κB inhibitor (SN50) and a JNK inhibitor (SP600125), but not a p38MAPK inhibitor (SB203580), inhibited the production of IL-1β, IL-6 and TNF-α by THP-1 cells treated with each of the five rL-hemolysins (rSph1, rSph2, rSph3, rHlpA or rTlyA) (**Figure 6**). However, in the rL-hemolysin-treated J774A.1 cells, the production of IL-6 and TNF-α was largely blocked by the JNK inhibitor but only partially blocked by the NF-κB inhibitor (**Figure 6**). As a control, *E. coli* LPS-induced IL-1β, IL-6 and TNF-α production was inhibited by all three inhibitors in both types of host cells, consistent with a previous report [47]. Furthermore, polymyxin B treatment of rL-hemolysins did not affect the results but proteinase K plus heating treatment made the rL-hemolysins lose the ability to induce the production of IL-1β, IL-6 and TNF-α. All these results suggest that the leptospiral hemolysins stimulate the production of proinflammatory cytokines through NF-κB and JNK pathways.

## Discussion

Inflammation is an essential component of an immune response against any microbial pathogen [49]. However, excessive inflammation can also result in tissue injury and physiological disorders [5], [50]. For instance, septic patients frequently die due to shock and multiple organ failure caused by the production of a large number of proinflammatory cytokines [51], [52]. Although there is a large diversity of clinical signs and symptoms among leptospirosis patients, a severe inflammatory response is common to all [1], [2], [12]. There is also a correlation between excessive TNF-α or IL-6 production and disease severity [6], [7], [9], [10], [15]. In the present study, we used protein arrays to determine complete cytokine profiles in the sera of leptospirosis patients as well as leptospire-infected mice. We then demonstrated that leptospiral hemolysins function as potent stimulators of proinflammatory cytokine production. Furthermore, we also showed that the leptospiral hemolysins induce the production of proinflammatory cytokines through TLR2-and TLR4-dependent NF-kB and JNK pathways.

The cytokine profile revealed that the levels of at least sixteen cytokines were significantly elevated in the leptospirosis patients’ sera, including the major proinflammatory factors IL-1β, IL-6 and TNF-α, and the major anti-inflammatory factors IL-4, IL-10 and IL-13 (**Figure 1**) [53]. This finding is consistent with the previous observation in leptospire-infected hamsters [11]. Golden Syrian hamsters and young guinea pigs are commonly used as experimental animals to study leptospirosis. However, there are no commercial protein arrays currently available for detecting cytokines in hamsters and guinea pigs. Mice are not fully susceptible to infection by leptospires [28], [33]. Previous studies reported a large diversity of susceptibility to infection with pathogenic *Leptospira* species in different mouse strains [54]. Leptospiral strains belonging to *L. interrogans* serogroup Icterohaemorrhagiae have been confirmed to cause acute lethal leptospirosis in young C3H/HeJ mice, a TLR4-deficient strain [55], with typical pathological changes such as hemorrhage, infiltration of inflammatory cells and vasculitis in the lung as well as diffuse inflammation and tubular necrosis in the kidney [29]–[31]. Although the C57BL/6 strain resists infection with pathogenic *Leptospira* species, mice with TLR2 and TLR4 double knock-out serve as a model of acute lethal leptospirosis [54], [56]. In the present study, we selected C3H/HeJ mice to detect serum cytokines after infection with *L. interrogans* serovar Lai strain Lai. However, in the leptospire-infected mouse sera, only seven cytokines including IL-1β, IL-6 and TNF-α were found to have elevated secretion (**Figure 1**). Interestingly, IL-17, a cytokine that promotes the release of IL-1 and IL-8 [57], was significantly elevated in sera from both the leptospirosis patients and leptospire-infected mice, which has never been reported before. The data demonstrated an evident diversity in serum cytokine profiles between leptospirosis patients and leptospire-infected mice, which might account for more severe clinical signs and symptoms in patients than in murine hosts with persistent leptospiral infection [2]–[4].

In China, the strains belonging to *L. interrogans* serogroup Icterohaemorrhagiae serovar Lai are responsible for at least 60% of leptospirosis patients [26], [28], [58], [59]. Compared to many other bacterial pathogens [60], [61], *L. interrogans* serovar Lai strain Lai possesses many hemolysin-encoding genes [18], suggesting that these hemolysins may play an important role in the pathogenicity of the spirochete. In previous studies, many recombinant hemolysins from different bacteria were used to determine their hemolytic and inflammatory cytokine-inducing abilities, in which the contaminating *E. coli* LPS was removed by such methods as Detoxi-Gel endotoxin-removing gel, and the possible residual trace of LPS was insufficient to cause an inflammatory response [62]. To obtain more exact and reliable results, in our study all the tested rL-hemolysins were pretreated with proteinase K plus heating or polymyxin B and used as controls to monitor possible contamination with *E. coli* LPS in the rL-hemolysin specimens.

Although the products of *hlyC* and *hlyX* genes of *L. interrogans* serovar Lai strain Lai are annotated as hemolysins [18], previous studies confirmed that hemolytic activity was absent from recombinant HlyC (also named TlyC) protein from *L. interrogans* serovar Pomona and recombinant HlyX protein from *L. interrogans* serovar Copenhageni [21], [63]. Our hemolytic assays demonstrated that the higher dosage (10 µg) of rHlyC or rHlyX from *L. interrogans* strain Lai showed hemolytic activity in the spectrophotometric hemolytic assay but no hemolytic rings on blood agar plates (**Figure 2D and 2E**). Since the blood agar plate hemolytic assay is generally considered as the gold standard for determining the hemolytic activity of putative bacterial hemolysins, we think that rHlyC and rHlyX may have no hemolytic activity during infection by the spirochete. Combined with the results of spectrophotometric measurements of hemoglobin release from sheep erythrocytes and hemolytic rings on sheep blood agar plates, the rSph1, rSph2, rSph3, rHlpA and rTlyA proteins from *L. interrogans* strain Lai were confirmed to have different degrees of hemolytic activity (**Figure 2D and 2E**). Interestingly, the mRNA levels of the hemolysin genes of *L. interrogans* strain Lai were relatively low in culture medium, but increased significantly when the leptospiral strain was co-incubated with THP-1 or J774A.1 cells (**Figure 3A**). The up-regulation of leptospiral hemolysin expression during infection of the host cells had been previously noted by us while investigating the expression of *L. interrogans* strain Lai transcripts [64]. In particular, only the Sph2 hemolysin was detectable by immunoblot assay in the supernatant of *L. interrogans* strain Lai cultured in EMJH liquid medium, whereas the spirochete secreted the Sph1, Sph2, Sph3, HlpA and TlyA hemolysins during co-incubation with THP-1 or J774A.1 cells (**Figure 3B**). These findings suggest that upon interaction with host cells, the spirochete upregulates the expression of these hemolysin-encoding genes and promotes secretion of the hemolysins into the host environment to facilitate infection.

LPS is a powerful inducer of proinflammatory cytokine production in hosts. Although leptospiral LPS is considered to be the major virulence factor for inflammation and tissue injury as well as an important stimulator to evoke protective immune responses during leptospirosis [57], [65], previous studies revealed that the biological activity of LPS from *Leptospira* species is much lower than those of LPSs from enteric bacteria [12], [66]. Viriyakosol *et al*. found that leptospiral LPS is not the only factor to induce the secretion of IL-6 and TNF-α in macrophages, because polymyxin B does not inhibit the inflammatory response [67]. In the present study, we found that all the five rL-hemolysins (rSph1, rSph2, rSph3, rHlpA and rTlyA) from *L. interrogans* strain Lai stimulated the secretion of IL-1β and TNF-α, while four of them (not rSph3) induced production of IL-6 (**Figure 4A**). Strikingly, the levels of cytokines induced by leptospiral rSph2, rHlpA and rTlyA were similar to that of *E coli* LPS. Thus, our data strongly support the notion that the leptospiral hemolysins, particularly the secreted hemolysins Sph2, HlpA and TlyA, are the major inflammation stimulators during leptospirosis.

Macrophages sense pathogens mainly through pathogen-recognition receptors such as the TLRs. In general, TLR4 acts as the receptor for bacterial LPS, while TLR1, TLR2, TLR5 and TLR6 recognize bacterial proteins such as flagellin and lipoprotein [68]. Previous studies demonstrated that the cytolysin/hemolysin of *V. cholerae* is recognized by TLR2 in host cells, while the hemolysins from several bacteria such as anthrolysin O, perfringolysin O and streptolysin O, are ligands of TLR4 [69], [70]. However, pneumolysin (α-hemolysin) from *S. pneumoniae* and seeligeriolysin O (α-hemolysin) from *L. seeligeri* are recognized by both TLR2 and TLR4 in human macrophages and renal epithelial cells [71], [72]. In this study, we demonstrated that among the five antibodies tested against TLRs (TLR1, TLR2, TLR4, TLR5 and TLR6), only TLR2-IgG and TLR4-IgG blocked the release of IL-1β, IL-6 and TNF-α in human and mouse macrophages induced by the rL-hemolysins (**Figure 5**). The results from the TLR2-or TLR4-or TLR2,4-deficient monocytes further reinforced the conclusion that the leptospiral hemolysins are recognized by both TLR2 and TLR4.

Ligation of TLR2 and TLR4 are known to initiate intracellular signal transduction through MyD88-dependent or-independent mechanisms to activate the NF-κB, JNK or p38MAPK pathways [48]. For instance, staphylococcal α-hemolysin was confirmed to induce IL-8 production in THP-1 cells through the NF-κB pathway [73]. Streptolysin O and listeriolysin O induce the production of TNF-α, IL-6 and IL-8 in mast cells and vein endothelial cells through the p38MAPK and NF-κB pathways [74], [75]. However, our results with signal kinase inhibitors suggested that the JNK and NF-κB pathways are involved in the production of IL-1β, IL-6 and TNF-α in both the rL-hemolysin-treated human and mouse macrophages (**Figure 6**), which differs from *E. coli* LPS that induces production of the three inflammatory cytokines through the NF-κB, JNK and p38MAPK pathways [47]. Taken together, our study demonstrated that the leptospiral hemolysins induce inflammatory reactions in human and mouse macrophages *via* TLR2-and TLR4-mediated JNK and NF-κB signaling pathways.
